# Utilizing Causal Loop Diagramming to Explore a Research and Evaluation Capacity Building Partnership

**DOI:** 10.3389/fpubh.2022.857918

**Published:** 2022-05-31

**Authors:** Rochelle Tobin, Gemma Crawford, Jonathan Hallett, Bruce Maycock, Roanna Lobo

**Affiliations:** ^1^Collaboration for Evidence, Research and Impact in Public Health, School of Population Health, Curtin University, Perth, WA, Australia; ^2^European Centre for Environment and Human Health, College of Medicine and Health, University of Exeter, Exeter, United Kingdom

**Keywords:** partnership, public health, research capacity, evaluation capacity, evidence-informed decision-making, systems thinking, causal loop diagram

## Abstract

The capacity to engage in research, evaluation and evidence-informed decision-making supports effective public health policy and practice. Little is known about partnership-based approaches that aim to build capacity across a system or how to evaluate them. This study examines the impacts of a research and evaluation capacity building partnership called the Western Australian Sexual Health and Blood-borne Virus Applied Research and Evaluation Network (hereafter, SiREN). SiREN aims to strengthen capacity across a system of clinical and medical services and government and non-government organizations. These organizations are connected through their shared aim of preventing and managing sexually transmissible infections and blood-borne viruses. To examine SiREN, systems concepts and methods were used. Data were collected from SiREN organizational documents (*n* = 42), a survey tool (*n* = 104), in-depth interviews (*n* = 17), a workshop and three meetings with SiREN stakeholders and used to develop two causal loop diagrams. Findings show engagement with SiREN was influenced by a complex interplay of contextual (e.g., organizational capacity) and process (e.g., presence of trusting relationships) factors. SiREN contributed to system level changes, including increased resources for research and evaluation, the development of networks and partnerships that led to more efficient responses to emerging health issues, evidence sharing, and sustainable research and evaluation practice. The use of causal loop diagrams enabled the identification of key leverage points that SiREN can use for continuous improvement or evaluation. The focus on how contextual factors influenced SiREN's ability to create change provides valuable information for researchers, policymakers or practitioners seeking to develop a similar partnership.

## Introduction

The capacity to engage in research, evaluation and evidence-informed decision-making supports effective public health policy and practice (1). Research and evaluation capacity building can be collectively defined as the intentional process of improving the motivation, knowledge, skills, and structures to engage in sustainable research and evaluation practice and apply research and evaluation evidence to decision-making (2–4). Evidence is acquired from multiple sources in public health, including research, evaluation, professional experience, surveillance data, and community perspectives, and then synthesized to guide decision making (5, 6). Capacity building can be theorized as a catalyst that releases potential from within individuals and organizations (7). For capacity building to be effective, it requires those involved to see the benefit and be committed to the process (7, 8). Strategies to build research, evaluation, and evidence-informed decision-making capacity in public health can target the individual, organization or system level. These strategies include training, tailored support, partnerships between researchers and decision-makers, the provision of resources (e.g., funding) and the development of infrastructure (e.g., research practice networks) (1–3, 9). Despite investment in capacity building strategies (7, 9, 10), little is known about developing and implementing them in different contexts, the kinds of impacts and outcomes they can achieve and the mechanisms by which change is achieved (7, 9, 11–14). A systems approach has been identified as a means to enhance understanding of capacity building initiatives (15). This paper describes a study using a systems approach to examine a research and evaluation capacity building project and inform its evaluation.

The capacity building project examined in this paper is called the Western Australian Sexual Health and Blood-borne Virus Applied Research and Evaluation Network (hereafter, SiREN). SiREN is a long-term partnership between sexual health and blood-borne virus (SHBBV) researchers, service providers and policymakers in Western Australia (WA) formally established in 2012 to strengthen evidence-informed policy and practice within the SHBBV sector in WA by developing research and evaluation capacity. A team of senior researchers coordinates SiREN within a large, global and highly ranked university (16). In Australia, the large majority of SHBBV research is generated by national centers located on the east coast and does not always address the specific SHBBV issues relevant to WA. The epidemiology of sexually transmissible infections (STIs) and blood-borne viruses (BBVs) in WA differs when compared to other parts of Australia (17, 18). This is in part due to the large land area, geographical isolation and differences in demographics. These factors have impacted on the availability of local SHBBV evidence for use by public health professionals. Compounding this, a recent survey of SiREN's stakeholders (individuals and organizations working to address SHBBVs) identified a perceived lack of research and evaluation capacity and insufficient access to relevant research as barriers to engaging in research, evaluation and evidence-informed decision-making (19). In response to WA specific needs, SiREN seeks to build stakeholder capacity to engage in research and evaluation and to build an evidence base relevant to WA SHBBV issues.

SiREN is embedded in a complex system composed of universities, clinical and medical services, and government and non-government organizations working toward the shared aim of preventing and managing STIs and BBVs in WA. The workforce composition is diverse and includes those in clinical, health promotion, peer-support, education, policymaking, and research-based positions. The system structure, activities and stakeholders constantly change in response to the social and political climate, variations in epidemiology, and developments in prevention and treatments (20, 21). The system is conceptualized as complex as it is composed of many interacting elements (individuals, organizations, relationships) that are dynamic and adapting, often in unpredictable ways (22, 23). SiREN can be considered as a series of ongoing events within the system that aims to influence the behavior and structure of the system, e.g., relationships, resources (24). SiREN aims to create change within the system through multiple strategies that include: delivering personalized research and evaluation support; providing tools, resources and evidence to guide program planning, research and evaluation; hosting a biennial research symposium; seeking grant funding; undertaking collaborative applied research and evaluation projects; facilitating and participating in research collaborations; and sharing the latest evidence, news and events with a network of over 430 individuals. The size of SiREN limits the scale of change; currently, it employs 1.4 full-time equivalent (FTE) staff as part of core funding and a further 4.0 FTE staff through additional grants. Additional descriptions of SiREN are available in previous publications (25–27).

Taking this complexity into account, a systems approach was employed in this research. Systems approaches are particularly suited to examining capacity building programs, like SiREN, that aim to create change across a system (15). This approach can also support the identification of indicators for ongoing monitoring and evaluation purposes (21). A systems approach can be used to understand a program by exploring the context in which it is implemented, the relationships between program and system elements, and patterns of change that occur over time (28, 29). Using such an approach can provide insight into how SiREN reshapes the system in beneficial ways including developing new capabilities, relationships and structures (28, 30). This study utilized causal loop diagrams, a type of qualitative systems modeling method that originated in the field of system dynamics (31, 32). This method uses word and arrow diagrams to visually represent stakeholder perspectives of the functioning of a system or program (33). They include feedback loops which are circular relationships between variables that can reinforce or balance change. Causal loop diagrams can provide insight into factors that influence a program's effectiveness and the kinds of changes it can achieve (34, 35).

While a solid evidence base supports partnerships and capacity building programs (4, 13, 36), little is known about how and in what ways they contribute to change (4, 36). Systems approaches to evaluation provide insight into the mechanisms of action and the identification of leverage points. These are crucial points within the system that can be influenced to effect change, enhance a program's effectiveness, and be used for monitoring and evaluation purposes (21, 37, 38). This study aimed to use systems concepts and methods to explore perceptions of (1) factors that influence engagement with SiREN, (2) the impacts and outcomes achieved by SiREN and the interactions between them, and (3) the use of causal loop diagrams as a method to understand SiREN and inform evaluation.

## Materials and Methods

This mixed-methods study used causal loop diagrams to examine factors that influence engagement with SiREN and the subsequent impacts and outcomes that occurred. The Consolidated Criteria for Reporting Qualitative Research (COREQ) checklist (39) guided reporting. Ethical approval was obtained for the study (approval number: HRE2017-0090). Informed consent was obtained from all subjects involved in the study. This study forms part of a larger project described in a previously published study protocol (26).

### Theoretical Framework

This research investigated how SiREN interacted with the system in which it is embedded and the impacts and outcomes that were achieved. Several different but overlapping areas of systems thinking were used (32, 40–42). Consistent with Checkland (39), the research study viewed a system as a mental model, built through drawing on multiple perspectives to facilitate understanding of the system. The study design used three principles from across the diverse field of systems methodologies and methods (40, 43), boundaries, perspectives and relationships. Boundaries determine what lies inside and outside a system (44) and are used to focus the inquiry (40, 44). In this study, the bounded system was the SHBBV virus prevention and management system, including SiREN and other organizations working to address SHBBVs in WA. Perspectives reflect an individual's point of view (40). The principle of perspectives acknowledges the plurality of views held by system stakeholders. Therefore, to enable a complete understanding of the system, multiple perspectives should be included (37, 45). In this study, a diverse range of views was sought along the spectrum of engagement with SiREN. Relationships are defined as causal connections between parts of a system (40). The principle of relationships focuses on how system variables interact and influence each other to achieve a purpose (46).

In this paper, relationships were explicated through causal loop diagrams (described above). This method is useful to describe how a program functions within the system it operates and enables program evaluation to move beyond individual project strategies to a more systemic view of changes over time (47, 48). Causal loop diagrams can act as a complexity sensitive theory of change (49–51). Causal loop diagrams have been used in other studies seeking to understand public health programs including prevention marketing (51), policy adoption (52), peer-based programs (21) and obesity prevention (35). However, they have not been used to evaluate a research or evaluation capacity building program (33). To date, the majority of studies exploring research and evaluation capacity building projects have applied more traditional approaches such as case studies and action research (8, 12, 53, 54). Lawrenz et al. (55) and Grack Nelson et al. (15) applied a complex adaptive systems lens to explore evaluation capacity building within a network. Other studies have applied a realist approach to research capacity building (7, 56). Cooke et al. (7) and Lawrenz et al. (55) concluded that complexity sensitive methods provide insight into how, and in what contexts, capacity building interventions work.

### Research Team and Reflexivity

During the time this study was undertaken, four research team members (RT, RL, JH, and GC) were employed by SiREN or members of the SiREN management team. The SiREN management team consists of five university-based staff with experience working in research, government, and policy involved in SiREN 's operational and strategic management. The research team had extensive experience in public health, qualitative research evaluation, and capacity building. All members of the team have experience working with, or within, community-based blood-borne virus organizations.

Most research team members are considered insider researchers (RT, RL, GC, JH) (57), with implications for data collection and analysis. In other ways they can be considered outsiders, e.g., they have not received support from, or partnered with, SiREN, and they are not currently working in a government or non-government organization. Insider researchers bring with them knowledge of the research problem and access to participants (58). In contrast, outsider researchers may notice aspects of the data that an insider may overlook as they appear ordinary to them (59–61). Researchers used a reflexive approach during data collection and analysis to identify and address bias, including regular meetings with the research team and reflective journaling (62). To validate findings, participants were invited to participate in a workshop to refine the study findings.

### Data Collection

Data were collected from SiREN organizational documents (*n* = 42) created between 2012 and 2020, a survey tool (*n* = 104) and in-depth interviews (*n* = 17) and used to inform the development of a draft causal loop diagram. Subsequently, the causal loop diagram was refined through a face-to-face workshop and three meetings with SiREN stakeholders (*n* = 4).

#### SiREN Organizational Documents

The following SiREN organizational documents (*n* = 42) were examined: biannual reports of activities and outputs (*n* = 18), reports evaluating SiREN activities (*n* = 6), needs assessment reports (*n* = 3), stakeholder emails describing impacts or outcomes of SiREN (*n* = 3), and stakeholder meeting minutes (*n* = 12). These documents provided an understanding of SiREN's activities, processes, impacts and outcomes.

#### Survey Tool

Every two years, the SiREN network is invited to participate in a needs assessment to inform SiREN activities and resource development. The SiREN network is a database of individuals across Australia with interest in SHBBVs. Summaries of relevant research and evaluation evidence, news, funding opportunities, and events are distributed *via* electronic mail. For this study, items were added to the needs assessment, and existing items were refined, using previous research and questionnaires (63–67). The survey tool was designed using Qualtrics survey-building software (68) and refined in consultation with three research team members (RT, GC, and RL). The final survey contained a combination of 43 open and closed questions, including factors that influence research, evaluation, and evidence-informed decision-making practices, details of engagement with SiREN, and the influence engagement had on practice. The survey was estimated to take 15 min. The survey was published as part of the study protocol (26). A link to the survey was emailed to WA-based SiREN network members (*n* = 204); just over 50% (*n* = 104) responded.

#### In-depth Interviews

In-depth, semi-structured, qualitative interviews were undertaken with SiREN partners and service users (*n* = 17), purposively selected stakeholders based on engagement with SiREN in the past 2 years. SiREN partner engagement was defined as one or more of the following: worked in partnership with SiREN to undertake a research or evaluation project; applied for research or evaluation funding with SiREN; or took part in the SiREN steering group. Participants were selected across different levels of engagement, including those who had engaged once to multiple times. The steering group is composed of key SiREN stakeholders from WA non-government organizations, government organizations, hospitals and research organizations who provide input into the strategic management of SiREN. Service user engagement was defined as having received tailored project planning, evaluation or research support, e.g., developing an evaluation framework. Participants were predominantly from WA-based government, non-government and research organizations, with the exception of one interstate research organization. Employment roles included managers, educators, project officers, clinical trainers, and researchers.

Interviews sought to explore participant experiences of engaging in research, evaluation and evidence-informed decision-making within the system and engagement with SiREN. The interview guide [see the published study protocol (26)] was developed in consultation with the research team (RT, RL, JH, and BM) and pilot tested with a SiREN staff member. Questions examined the contextual factors influencing research, evaluation and evidence-informed decision-making practices, details of engagement with SiREN, and how and in what ways engagement with SiREN influenced practice.

Twenty-two individuals were invited *via* email to participate. Three did not respond to the invitation and two declined citing conflict of interest as SiREN's main funder employed them. Face-to-face interviews were undertaken with metropolitan participants at their workplace and *via* telephone with regional and interstate participants. The duration of the interviews ranged from 30 to 90 min. Interviews were digitally recorded, transcribed verbatim and reviewed for accuracy by RT. Transcripts were not member checked.

### Draft Causal Loop Diagram Development

To develop the causal loop diagram, data from organizational documents, surveys and interviews were open-coded using NVivo 11 software (69) by RT similar to the grounded theory-informed approach recommended by Kim and Andersen (70). Coding was guided by the areas addressed in survey and interview questions including contextual factors that influence research, evaluation, and evidence-informed decision-making practices, factors that affect engagement with SiREN, and outcomes achieved by SiREN. Data were coded into categories until no new variables were identified and superordinate categories emerged. The second phase of coding identified system variables, causal relationships, feedback loops and time lags to inform the structure of the causal loop diagram. As part of this process, emerging variables and relationships were discussed and refined in consultation with members of the research team (RT, RL, JH, BM).

To link the causal loop diagram variables and relationships to their data source, a reference table modified from Kim and Andersen (70) was created using Microsoft Excel (Version 2105). This table included all variables, their relationships and supporting data. An example is provided in Table 1.

**Table 1:** Coding table example.

**Variable**	**Effect variable**	**Relationship type**	**Supporting data and source**
Trust built	Engagement with SiREN	Positive	*(SiREN's) got a nice connection with NGOs (non-government organizations), and I think there's a lot of trust between NGOs and the Government Department of Health with SiREN. And I think that helps facilitate it (engagement) as well*. Source: Interview (P14).

Identified variables and their relationships were transformed into a causal loop diagram using Vensim (71), a software program used for creating and presenting causal loop diagrams. The process of data collection, analysis and diagram building occurred concurrently.

### Validating the Causal Loop Diagram

A 2-h workshop was held to validate the causal loop diagram. Participatory processes strengthens the validity of the causal loop diagrams and was used in similar studies (34, 72). In-depth interview participants (*n* = 17) and SiREN management team members (*n* = 5) were invited by email to participate. Workshop participants included in-depth interview participants (*n* = 5), SiREN management team (*n* = 3) and an observer from the research team (BM).

The workshop was facilitated by a researcher (RT). In the workshop, the facilitator provided a brief overview of systems thinking, guidance on how to interpret causal loop diagrams and a description of the diagram. Questions were then posed to the group including: if the diagram reflected their experience of SiREN, if there were any aspects not represented and if they had any comments on the terms used to describe the variables. Participants were seated around a square table, and in the center of the table was a laminated copy of the diagram (A0 size) and whiteboard markers. This format enabled the alteration of the variables and relationships as the group discussed them. The role of the management team in the validation process was not to provide their perception of the changes that SiREN had achieved but to support the interrogation of the diagram by asking questions, for example, seeking clarification on the meaning of variables and the nature of the relationships between them.

Following the workshop, three meetings of 30–60 min were held. Two meetings were held with individual members of the management team who could not attend the workshop and a meeting with members of the research team (*n* = 4) to refine the diagram. RT further developed diagrams in consultation with the research team to ensure they were able to be easily interpreted in published form and when the process of writing revealed new relationships and variables. One of these changes involved splitting the diagram into two, leaving the central variable of engagement with SiREN in both diagrams. This enabled the processes that influence engagement and the subsequent impacts and outcomes that occur to be clearly depicted.

## Results

Two causal loop diagrams illustrate 1. factors affecting engagement and 2. impacts and outcomes. Diagrams are presented, followed by a table that describes the corresponding variables in alphabetical order. An explanatory narrative supports the diagrams and table, and deidentified participant quotes illustrate findings. The narrative discusses diagram variables and relationships under related topic headings.

To read the diagrams, select a variable of interest and follow the causal connections. Relationships between variables are either positive (represented as “+”) or negative (represented with “–”). The system variables and relationships join to form feedback loops. Feedback loops illustrate circular cause and effect relationships that can be reinforcing where they amplify change (represented with an “R”) or balancing where they attenuate change by driving change in the opposite direction from where it started (represented with “B”) (48). Time delays (represented by a “//”) occur where there is a delay in a change occurring (48).

### Engagement

Analysis identified two types of engagement, transactional and synergistic. These are important determinants of the kinds of impacts and outcomes that were achieved. Transactional engagement was identified as brief, addressing a specific question within one or two interactions with SiREN. Examples of transactional engagement included support for writing a conference abstract or refining an existing evaluation tool. Transactional engagement led to increased research and evaluation confidence, knowledge and skills. Synergistic engagement was identified as occurring over multiple interactions with SiREN across an extended period of time, (e.g., months, years) and led to the development of trusting relationships. It involved both parties combining their knowledge to address research and evaluation issues, such as developing a program evaluation plan or research proposal and had the potential to lead to all identified impacts and outcomes.

**Figure 1:**
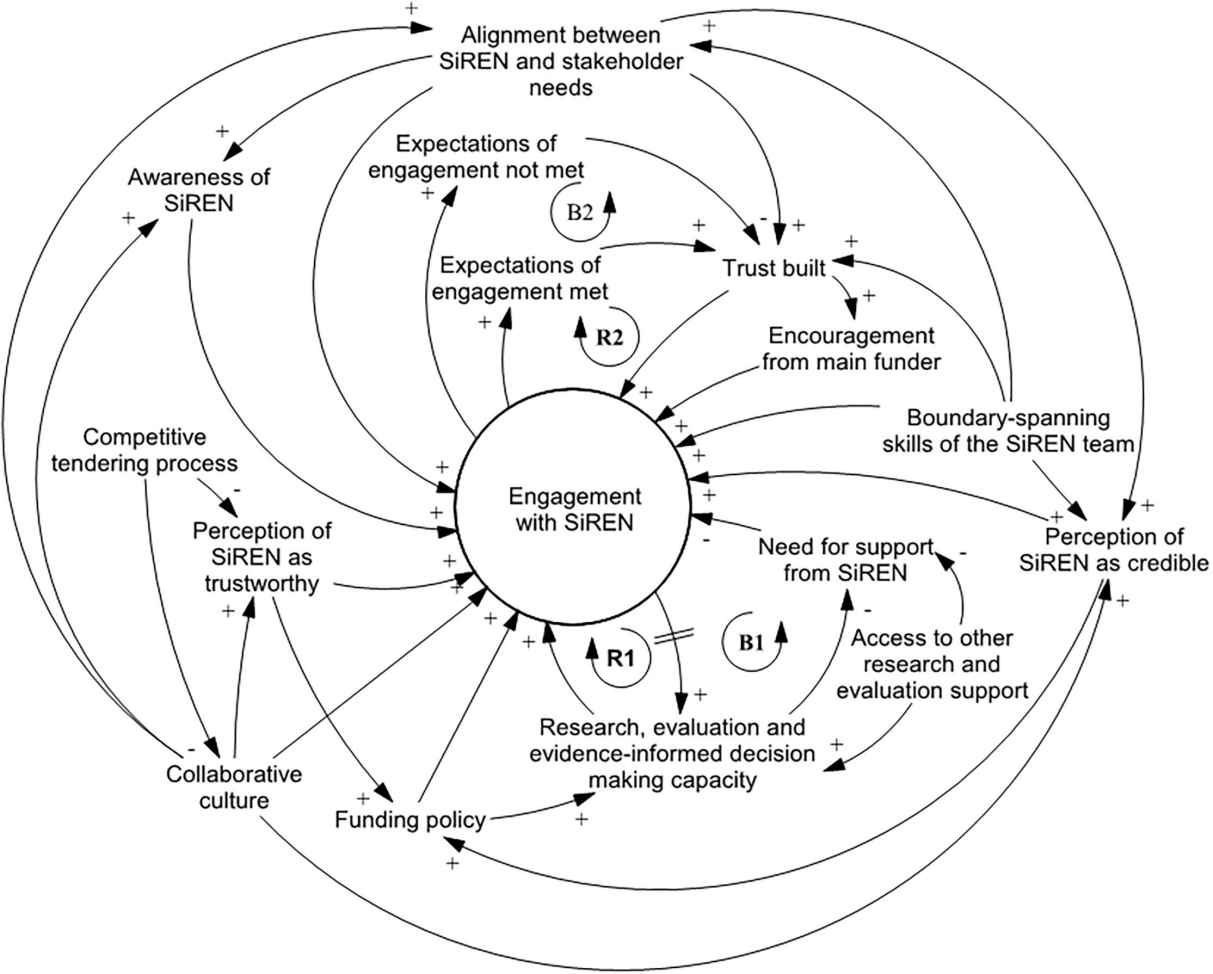
Causal loop diagram depicting factors that influence engagement with SiREN.

The first causal loop diagram (Figure 1) illustrates factors that influenced engagement with SiREN. Diagram variables are defined in Table 2. The diagram indicates that engagement with SiREN is dynamic and changed in response to factors within the control, (e.g., presence of trusting relationships) and outside SiREN's control, (e.g., organizational evaluation capacity).

**Table 2:** A description of variables that influence engagement with SiREN.

**Variable**	**Description**
Access to other research and evaluation support	Support available beyond the support provided by SiREN, e.g., relationships with other research centers.
Alignment between SiREN and stakeholder needs	SiREN's services were compatible with the research, evaluation, and evidence-informed decision-making needs of stakeholders.
Awareness of SiREN	Stakeholders understood what SiREN is and the kinds of services and support it can offer.
Boundary-spanning skills of the SiREN team	Ability to build relationships and facilitate learning across diverse groups (73), e.g., research and service delivery.
Collaborative culture	Stakeholders had a history of working together, as well as with SiREN team members, to address SHBBV issues.
Competitive tendering process	Organizations needed to compete for funding from the main funding body.
Encouragement from main funder	The main funder encouraged funded organizations to engage with SiREN when they require research and evaluation support.
Engagement with SiREN	Occurred when a partner or service user participated in a SiREN advisory group; partnered with SiREN to undertake research, evaluation or apply for a grant; or received program planning, research, evaluation, or evidence-informed decision-making support.
Expectations of engagement met	When SiREN met partner or service user expectations of what SiREN will do, e.g., develop an evaluation tool.
Expectations of engagement not met	When SiREN did not meet partner or service user expectations of what SiREN will do.
Funding policy	The main funding body stipulated that some funded organizations must engage with research organizations for research and evaluation purposes. Funded programs were contractually obligated to be evaluated.
Need for support from SiREN	The need for support from SiREN arose when an individuals or organization's capacity did not meet their requirements of their role.
Perception of SiREN as credible	Stakeholders perceive the information provided by SiREN as reliable.
Perception of SiREN as trustworthy	Stakeholders felt that information shared with SiREN will be kept confidential. This view can be held because of an interaction with SiREN or because of SiREN's reputation.
Research, evaluation and evidence-informed decision-making capacity	The motivation, knowledge, skills, and resources to undertake research and evaluation and apply evidence to decision-making (2–4).
Trust built	Developed through repeated interactions over time. Trust enabled partners and service users to know SiREN will act in a trustworthy way (74).

#### Existing Relationships Act as a Springboard

The presence of a *collaborative culture* within the system increased engagement with SiREN. This culture predated SiREN and was traced back by interview participants to Australia's partnership-based response to the HIV epidemic (75). Participants reported that this legacy of collaborative working continues to influence how connected they are. In addition, the SiREN management team had a decades-long history of working with, and within, government and non-government organizations. The relationships formed during this time included those of research partners, colleagues, and friends. These relationships acted as a springboard to generate awareness of SiREN, support its credibility, and develop the partnerships and networks that underpin its approach:

*(SiREN is) a reliable source of support, it comes from the SiREN team as I said, I suppose, being embedded within (the University), those past relationships that I, we, the sector has had with (the University) over many, many years. (P10)*

#### Support From Funders

The funding environment had a dual effect on engagement. On the one hand, *funding policy* increased engagement as the main funding body encourages funded organizations to actively work with research-based organizations, like SiREN, for research and evaluation purposes. On the other hand, the main funding body recently transitioned its funding model from a preferred service provider status to a *competitive tendering process*. This model resulted in some organizations competing with one another for funding. Participants suggested this transition had a detrimental effect on the *collaborative culture* and resulted in a lack of clarity regarding whether SiREN could be trusted to provide confidential support to all applicants for competitive funding.

#### Perceptions of SiREN

Engagement increased when stakeholders perceived SiREN to be trustworthy and credible. Credibility was enhanced by SiREN's association with the University, which gave SiREN source credibility (76) and its relationships to other organizations working within the system which provided credibility by association (77). Other factors that enhanced perceived credibility included the visibility of SiREN, (e.g., presentations at events and publications) and the view that SiREN is a “storehouse” of knowledge for the sector:

*I think it was the backing of a university… that I think makes (SiREN) a really credible source for that type of advice… it's SiREN acting as more of the point of contact for lots of other organizations that may have contacted them for the same thing. (P4)*

#### The Relationship Between Capacity and Need for Support

*Research and evaluation capacity* and *the need for research and evaluation support* was dynamic and varied across the system. Participants identified a range of factors that influenced their capacity to engage in research and evaluation including: level of knowledge and skills, attitudes and values, accessibility of target groups and data, access to resources, (e.g., funding and time), requirements of funding bodies, and the availability of internal and external research and evaluation support. Participants required the capacity to engage in research and evaluation to engage with SiREN, e.g., through time or support from management. Engaging with SiREN increased *research and evaluation capacity*. In some cases, this boosted *engagement with SiREN* as awareness of, and ability to, engage in new research and evaluation opportunities (e.g., developing new evaluation methods, research projects) increased [Figure 1, reinforcing loop 1 (R1)]. This was explained by a service user who had recently commenced a research project in partnership with SiREN:

*(SiREN team member has) been encouraging me to find these sort of research projects, you know, and so I'm starting to kind of now see opportunities which is great… and I know that when I take that step I'll have the support I need. (P6)*

However, when *research and evaluation capacity* increased due to receiving support from SiREN, it could also lead to a decrease in engagement. This is because the need for *research and evaluation support* decreased, leading to a reduction in *engagement with SiREN* as service users felt they had the resources and skills to meet the requirements of their role [Figure 1, Balancing loop 1 (B1)]. A non-government organization staff member reflected on why they had not engaged with SiREN since receiving support to develop a logic model program plan:

*I've been able to keep the ball rolling and rather confidently go through my project… Knowing I'm doing the right thing that I'm supposed to be doing in exactly the right way, with the knowledge I'm supposed to have that's up to date. (P5)*

*Need for support from SiREN* also decreased when participants had *access to other research and evaluation support*, e.g., a new research officer working within their organization.

#### The Effect of Trust

When trusting relationships were built between SiREN and its partners or service users, it increased engagement [Figure 1, reinforcing loop 2 (R2)]. Because of the reinforcing effect between trust and engagement, there was increased potential for impacts and outcomes. Trust was identified in analysis as a leverage point due to its central role in strengthening relationships and its potential to enhance the impacts of SiREN. The development of trust was a social process whereby partners and service users learn through experience that SiREN will act reliably (74):

*I think it's about showing credibility, following through with promises. So, saying they'll do something and actually doing it. (P9)*

As highlighted in the quote, credibility, integrity, capability, and meeting expectations were important components of trust related to SiREN. Trust was dynamic and could be affected. For example, as reflected in Balancing Loop 2 (Figure 1, B2), one participant reported that their *expectations of engagement were not met*. In this instance, engagement decreased but did not cease indicating that *trust* was reduced but not lost.

#### Positioning of SiREN

Another leverage point was the *boundary-spanning skills of the SiREN team which* boosted engagement. These qualities were attributed, in part, to the past and current experience of the team working across research, clinical, government and non-government organizations. These experiences furnished team members with an understanding of how to undertake and support research and evaluation in policymaking and service delivery contexts and how to communicate with diverse groups of people. Participants described these qualities as being approachable, understanding, having expertise, and supporting the exchange of knowledge:

(*SiREN Team Member was) so forthcoming and it was so quick for her to identify where I was at and was easy for me to understand where she's at, that compatibility of how we could share knowledge. (P12)*

*The boundary-spanning skills of the SiREN team* facilitated *alignment between SiREN activities and stakeholder needs*. Boundary-spanning skills supported the transfer of knowledge (78) from stakeholders to SiREN. SiREN subsequently used this knowledge used to align its services to their research and evaluation needs. The alignment process was aided by SiREN's governance structure, as both the management team and steering group members contributed their understanding of the system into decisions of how SiREN delivered its services. Other processes that increased *alignment between SiREN and stakeholder needs* included a biennial stakeholder needs assessment and a research priority-setting process. The needs assessment sought to understand the research and evaluation needs of stakeholders to inform SiREN activities. The research priority-setting process involved working with the sector to establish key research priority areas and support the development of collaborative research grant applications to address agreed topics. SiREN also informally exchanged knowledge with stakeholders at meetings and events which informed alignment. Alignment strengthened *trust* between SiREN and its partners and service users and provided SiREN with the insight required to develop solutions to research and evaluation challenges:

*I do feel that the sector has grown. I feel that SiREN's grown, and I think they've actually grown together… (SiREN) understanding the sector more, and the challenges that come, but also having some great ideas on ways to deal with those challenges as well. (P9)*

### Impacts and Outcomes

**Figure 2:**
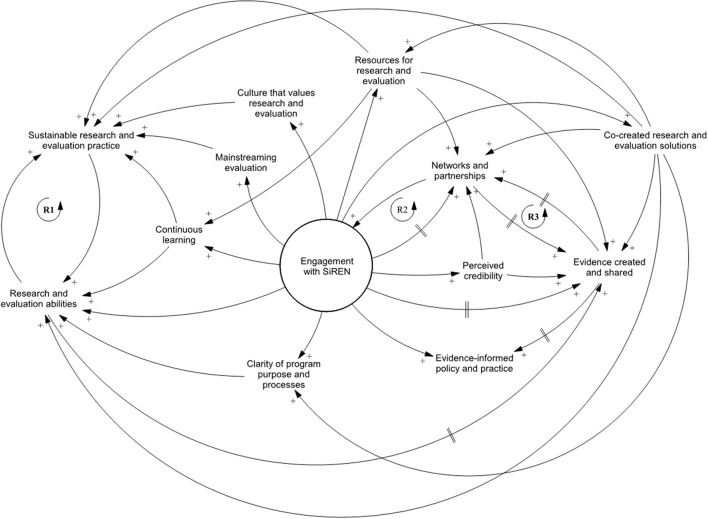
Causal loop diagram of impacts and outcomes.

The second causal loop diagram (Figure 2) explores the impacts and outcomes that have resulted from engagement between SiREN, its partners and service users. The diagram shows that an occurrence of an impact or outcome does not mean an end point has been reached; rather it is feedback into the system as an input and continues to create change. The variables for this diagram are defined in Table 3.

**Table 3:** A description of impact and outcome variables.

**Variable**	**Description**
Clarity of program purpose and processes	Understanding what a program is aiming to achieve, how it will achieve it, and how it fits within the broader SHBBV prevention and management system.
Co-created research and evaluation solutions	SiREN, its partners and/or service users combined knowledge to co-create research and evaluation solutions, e.g., evaluation method or a research grant application.
Continuous learning	SiREN provided a range of opportunities to engage in ongoing learning, e.g., workshops, online resources, post graduate research.
Culture that values research and evaluation	Value the contribution that research and evaluation makes to their practice and is open to participating in new research and evaluation opportunities.
Engagement with SiREN	Engagement occurred when a SiREN partner or service user participated in a SiREN advisory group; partnered with SiREN to undertake research, evaluation or apply for a grant; or received program planning, evaluation or research support.
Evidence created and shared	SiREN worked collaboratively to create and share an evidence base that is relevant to local issues, e.g., journal article or report.
Evidence-informed policy and practice	Involves combining the best available evidence from research and evaluation, experiential knowledge and contextual factors to inform decision-making (5).
Mainstreaming evaluation	Integrating evaluation as part of routine practice (79), e.g., the development of evaluation plans.
Networks and partnerships	Formal and informal relationships between researchers (including SiREN), service providers and/or government to create and share evidence and knowledge.
Perceived credibility	Perceived the credibility of the evidence created or their program and/or organization increased after receiving support from SiREN.
Research and evaluation abilities	The confidence, knowledge and skills to undertake research and evaluation and apply evidence to decision-making.
Resources for research and evaluation	Resources included financial and human resources, e.g., research grant funding or opportunities for postgraduate research students.
Sustainable research and evaluation practice	Research and evaluation capacity is maintained or increased over an extended period.

Impacts are defined as short-term changes that generally occur before outcomes, such as increased research and evaluation confidence, knowledge and skills. Outcomes are longer-term changes, an example being the application of evidence to policy and practice decision-making (80).

#### Clarity, Ability and Credibility

When SiREN provided program planning and evaluation support, a logic model program plan was often developed that outlined objectives, strategies, and evaluation measures. Several participants explained the process of creating this plan increased clarity around their program:

*That was the biggest thing that I got out of it (working with SiREN), was having that really clear understanding of this is exactly what I'm trying to do, and this is how I need to do it. (P5)*

Engagement with SiREN and *clarity of program purpose and processes* increased *research and evaluation abilitie*s at an individual level. Some participants described losing confidence in their evaluation skills in their initial engagement with SiREN as they developed more comprehensive knowledge and capability. The ongoing and flexible support provided by SiREN provided individuals with an opportunity to engage in *continuous learning*. This iterative, action-oriented process of learning and doing while supported by SiREN, enabled participants to put new knowledge and skills into practice and re-build their confidence:

*I had thought that I had a handle on exactly what I was trying to achieve in my project at that particular time and how I would measure it. It wasn't until I went through this formal process of having to strip it back, that I realised that maybe I didn't quite have the handle that I thought I had. (P5)*

Several participants reported that engaging with SiREN increased the credibility of their program and research or evaluation findings. This increased confidence to share their work at conferences and events and work in partnership with other organizations.

#### Building Sustainable Research and Evaluation Practice

SiREN contributed to building *sustainable research and evaluation practices* by increasing r*esearch and evaluation abilities* [Figure 2, reinforcing loop 1 (R1)]. As abilities developed, individuals and organizations were more likely to engage in research and evaluation activities, thus increasing research and evaluation practice sustainability. SiREN has also supported *sustainable research and evaluation practices* by developing a *culture that values research and evaluation*. Participants spoke about how they placed more value on evaluation and research due to engaging with SiREN. One participant reflected on how they now felt comfortable taking risks, e.g., pursuing a new research project, knowing they had the support of SiREN. This growth in research and evaluation culture built sustainability by increasing research and evaluation activity as service users saw the benefits it brought to their work:

*It's not something you just tag on the end of something. I've learned a lot about the importance of evaluation… now I want to spend more time on evaluation… But it's not because I have to do it, it's because I need to do it. Because at the end of the day, that's so important for funding… I can see the impact that this training could really have if I evaluate it properly*. (P11)

SiREN has also supported *sustainable research and evaluation practice* by working with individuals and organizations to develop logic model program plans that contributed to *mainstreaming evaluation* in their organizations. Furthermore, it provided c*ontinuous learning opportunities* such as the supervision of postgraduate research students; the provision of online resources such as evaluation toolkits; and the delivery of personalized research and evaluation support. These changes begin at the individual level. Over a period of several years, SiREN has been able to build capacity within multiple organizations leading to these changes being evident across the system. However, the dynamic nature of the system, e.g., funding and staffing changes, can disrupt this.

#### Relationships and the Co-creation and Sharing of Evidence

SiREN worked with its partners and service users to *co-create research and evaluation solutions*. These solutions included creating evaluation plans, designing evaluation tools, and developing research grant applications to address gaps in the evidence base. As part of this process, knowledge of contextual factors, (e.g., target group, setting) and research and evaluation methods, (e.g., survey development) was combined to develop practical solutions:

*Everything we did, we tested and then (SiREN staff member) and I would have a discussion about it, and then… so, it went through several changes before we got an assessment tool (evaluation survey) ready to use… It meant I knew the assessment tool was going to be appropriate. The process was rigorous, we had thought of everything. (P12)*

The ability of SiREN to connect stakeholders from diverse backgrounds to address challenges is an indicator of effectiveness at the system level (77). Between 2012 and 2020, SiREN has led and supported over 14 collaborative research and evaluation projects that have bought together researchers, practitioners and policymakers from around Australia, including a large national competitive grant. This has generated $1.5 million in additional financial *resources for research and evaluation* within the system. SiREN acted as a relationship facilitator by connecting researchers across Australia with WA based organizations to support the development and implementation of applied research projects. The benefits of SiREN's connections were noted by one of its research partners:

*The thing that's probably allowed us to consider WA more often, has been that not only having SiREN, but people who get that approach (applied research) and can kind of be the people that work directly with some of the agencies… what it means is it is a much more genuinely and true collaborative relationship… it's just really difficult to maintain a true collaborative project with that kind of distance. (P15)*

The development of *networks and partnerships* has a reinforcing relationship with engagement; increased connections within the system led to new stakeholders engaging with SiREN [Figure 2, reinforcing loop 2 (R2)]. *Networks and partnerships* also had a reinforcing relationship with creating and sharing evidence [Figure 2, reinforcing loop 3 (R3)]. A lack of contextually relevant research is acknowledged as a barrier to evidence-informed decision-making (81). To address this, SiREN has supported creating an evidence base relevant to WA's SHBBV unique priorities and challenges. This was achieved through two main strategies: building the capacity of stakeholders to generate research and evaluation evidence; and participating in, and facilitating collaborative partnerships between researchers, service providers and policymakers to create and share evidence. Knowledge sharing occurred at a system level and was facilitated by disseminating evidence, (e.g., learning resources, findings from research and evaluation projects) through its website, social media accounts, video case studies, regular electronic communications to its member network, (e.g., evidence summaries), and biennial 2-day research symposium. In addition, SiREN supported knowledge sharing by providing training, support, and resources to build confidence and skills of the SHBBV workforce to share research and evaluation findings at conferences and other fora. Table 4 presents a summary of SiREN's tangible evidence and capacity building outputs which support the study findings.

**Table 4:** Summary of SiREN's evidence and capacity building outputs from 2012 to 2020.

**Activity**	**Output**
**Evidence building and translational research**	
Peer reviewed journal articles	48
Reports / other publications	17
Conference abstracts, presentations, workshops, or posters	57
**Workforce development and capacity building**	
Hours of tailored research and evaluation support provided to 23 organizations	1,137
Events delivered or co-facilitated by SiREN	32
Post graduate students supervised (Honors, Masters and PhD)	33

#### The Application of Evidence to Decision-Making

Evidence created by SiREN and its stakeholders has been used by government to inform policy decisions at both the state and national levels. For example, SiREN recently completed an evidence review which informed the development of strategies that guide the response to SHBBV issues across the state of WA (82). In addition, organizations have used evidence created by SiREN to inform how their services are delivered. An example is the use of a report written by SiREN (83), which a participant described:

*Staff refer to it (a report produced by SiREN) to inform the work they're doing around culturally and linguistically diverse communities… So that report certainly drove both local programs but also I think a lot of the advocacy work of WA to the rest of the country. (P14)*

Another way SiREN has supported evidence-informed decision-making is by assisting organizations to evaluate their programs. Evaluation findings were then combined with other sources of evidence, (e.g., research and experiential knowledge) to inform program delivery. This was explained by a manager whose non-government organization had received support to plan and evaluate each of their programs, including support to deliver focus groups:

*We've increased the amount of evaluation that we've done to justify being able to do the things that we need to do to increase the services. We've got that (new service), and that's a genuine, direct result of the research that's been out there around the importance of taking services to people and also from us doing focus groups. (P9)*

## Discussion

A systems approach explored how and in what ways a research and evaluation capacity building project (SiREN) supported research, evaluation, and evidence-informed decision-making capacity within a system focusing on the prevention and management of STIs and BBVs (the system). Situating SiREN within the system enabled the research to address gaps in the existing capacity building literature. Including examining how contextual factors interacted with SiREN's ability to create change, how SiREN contributed to change across multiple levels, and the kinds of change it achieved (14).

### Synergistic Engagement to Create Change

Synergistic (extended) engagement between SiREN, its service users and partners led to more impacts and outcomes than transactional (brief) engagement. While these different types of engagement are not depicted in the causal loop diagrams, describing them provides insight into the kinds of changes different capacity building strategies can achieve (7, 84). In the partnership literature, synergy occurs when partners combine their knowledge, skills and resources to develop effective solutions (77). Synergy is based on trusting relationships (85), which, once established, lead to more significant change. In this study, the effects seen from synergistic engagement are attributed to the presence of trust, adapting support to the service user's needs, and/or providing them opportunities to learn by doing. This aligns with theories of capacity building, highlighted in the introduction, that emphasize the importance of those involved being committed and seeing value in the capacity building process (7, 8). While this study and others (84, 86) acknowledge the benefits of transactional engagement strategies as part of a multi-component approach to building capacity, synergistic engagement had the ability to create sustainable change, (e.g., from increased individual research and evaluation skills to sustainable research and evaluation practice). These findings align with recent studies (7, 55, 84, 86), which found strategies that are needs-based and provide practical opportunities to apply learnings are an effective and meaningful way to build capacity.

### Leverage Points

One of the most valuable insights gained through the use of causal loop diagrams was identifying key points of influence within the system. The development of trusting relationships between SiREN, its partners, and service users was identified as a point essential to SiREN's success. Trust had a reinforcing effect on engagement with SiREN [Figure 1, reinforcing loop 2 (R2)]. While trust is widely accepted as a fundamental component of effective partnerships (36, 77) and research capacity building efforts (7, 87, 88), it has not been explored within the evaluation capacity building literature (89). This research suggests that development of trust in evaluation capacity building parallels the research capacity building and broader partnership literature. The findings indicate trust was predicated on credibility, reliability, and power-sharing to define problems and shape solutions (85, 90). The role these factors played was evident in the trust-building effects of meeting expectations, boundary-spanning skills of the SiREN team, and the collaborative processes of aligning SiREN to stakeholder needs. Identifying leverage points enables action on these points of influence to strengthen its functioning (91).

### Change Across the Individual, Organizational, and System Level

There is a need for capacity building programs to focus on change at a system level (e.g., creation of shared research priorities, priorities of funders, partnerships, and sustainability) (92). An evaluation of SiREN, undertaken 2 years after initial funding, identified individual-level improvements to research and evaluation attitudes, knowledge, skills and confidence (25). For the present study, data were collected up to 8 years after SiREN was established and showed these individual-level changes had continued and identified further changes evident across individual, organizational and system levels. Organizational level changes were co-created research and evaluation solutions, mainstreaming evaluation, and evidence-informed decision making. System level changes included increased resources for research and evaluation (e.g., funding), the development of networks and partnerships that led to more efficient responses to emerging issues (e.g., collaborative research priority setting), evidence sharing, and sustainable research and evaluation practice. While many system level changes begin at the individual level (e.g., support to undertake a research project), they can reverberate across the system over time when they occur through synergistic engagement. This “ripple effect” theory has been identified previously in the research partnership literature (88). The sustained investment in SiREN by its primary funder provided the resources to achieve these valuable longer-term changes. Supported by this research is the need for greater awareness that capacity building initiatives may not yield outcomes in the first few years. This finding is important to manage stakeholder expectations of what can be achieved and identify appropriate evaluation time points. This is a valuable consideration for groups interested in implementing capacity building initiatives, particularly in negotiating key performance indicators with funding organizations or the timing of evaluation.

The authors acknowledge that SiREN is just one of many influences on research and evaluation practices within the system. While SiREN elicited meaningful change at an individual and organizational level, which has rippled outwards to system level change, its ability to produce change directly at the system level is limited by its scope and size. Adding to this challenge is that complex systems exist in a permanent state of change (93). In this system, there is a perpetual movement of staff in and out, there are changes to funding, and epidemiological variations occur requiring new resources and evidence to respond. There is need for continuous capacity building in public health (94), yet how to achieve sustained change from capacity building strategies requires further exploration (84). SiREN's continued investment in aligning its services and resources to the needs of stakeholders support its ability to address emerging changes. Furthermore, its contribution to embedding evaluation as part of regular practice in the system and the continuous learning opportunities it provides increase sustainability by ensuring that the impacts of its capacity building strategies efforts do not diminish over time (2). Therefore, system level capacity building projects need to be flexible and responsive to change within the system they operate and approach capacity building as a continual process rather than an end point.

Many of the impacts and outcomes achieved align with what is widely known in the capacity building literature, e.g., changes to knowledge and skills, the establishment of networks and partnerships (2, 86, 87). However, unexpected changes were also identified, including increased clarity amongst SiREN service users of their program purpose, processes and credibility of programs. Identifying unanticipated outcomes demonstrates the benefit that a systems approach contributed to understanding SiREN's changes. Systems approaches go beyond measuring the extent to which pre-determined objectives or goals are met, which is a common end-point in more traditional evaluation approaches. The detection of unexpected outcomes suggests the evaluation of capacity building projects can be strengthened through approaches that are sensitive to their complexities (43).

### Development of Practical Indicators

One of the aims of creating the causal loop diagrams was to gain an in-depth understanding of SiREN to inform the subsequent development of a comprehensive evaluation framework. Causal loop diagrams can support the identification of high quality and useful indicators (21). Insights from this study have since been used to develop specific indicators to monitor SiREN's processes, impacts and outcomes. For example, the presence of trusting relationships has been identified as an important indicator due to its reinforcing effect on engagement. In addition to an evaluation framework, a questionnaire for SiREN service users was subsequently developed based on findings (described in a forthcoming publication).

### Strengths and Limitations

The use of causal loop diagrams and supporting quotes provided credible explanatory links between SiREN and changes that occurred (95). In addition, the causal loop diagram illustrating factors that influence engagement with SiREN strengthens understanding of how contextual variables interact and affect implementation and effectiveness. Explaining contextual factors and their relationship to the functioning of SiREN avoided over or under-stating causality and ensured key elements that influence functioning were not obscured.

In public health, many causal loop diagram studies are created only by the researcher team, without input from stakeholders (96). Collaborative model building processes can help stakeholders overcome difficulties with interpretation (97), develop a shared understanding of how systems variables and relationships drive change (21) and create consensus on how to address the issue illustrated by the diagram (97). The process and value of the collaborative model building was not assessed in this study. Most protocols for developing casual loop diagrams focus on the early stages of group model development (98, 99). Guidance on validating diagrams at later stages of development is limited to individual interviews (33, 100). Refining diagrams using individual interviews may be better at clarifying and capturing different perspectives when compared to group methods (101). Future causal loop diagram studies could examine group processes of model development at the later stages of model development.

As staff employed by SiREN's primary funder declined to participate, the study findings do not include their perspectives. This may mean that some impacts and outcomes were not identified. As with any modeling, simplification was required. Not all feedback loops were reported for the diagram depicting impacts and outcomes as they were too numerous and would overcomplicate the presentation of study results. Instead, the diagrams are supported through additional detail provided by the narrative description.

As members of the research team are involved with SiREN, social desirability bias may have occurred during data collection (102). This was reduced by utilizing a variety of data collection methods, providing participants with assurances of confidentiality, probing to clarify in-depth interview responses, and discussing data collection processes with the SiREN team (102). Several strategies addressed the limitations associated with insider research and a single researcher collecting data and conducting primary analysis. Trustworthiness was increased through data triangulation, reflective journaling and regular meetings with the research team during data collection and analysis to discuss and refine emerging findings (103). During these meetings, a team member who was not involved in SiREN was present to enhance objectivity (103). In addition, diagram elements were linked to data sources in a reference table (70), and the diagram was validated with participants, a form of member checking (104, 105). The diagram was modified for publication after this validation process. The changes were based on data collected and included splitting the diagram into two and adding additional variables and relationships. These changes were intended to increase the accuracy of the diagram and support its interpretation in published form. Refining diagrams after data collection has ceased has been used in previous studies and aligns with good model building practice (106). Furthermore, developing “reader friendly” casual loop diagrams requires considering how the diagram functions as an effective tool for communicating findings (96). However, changes were not checked with original participants, which may have reduced the trustworthiness of the diagrams. Data collection occurred up to 2 years after some participants engaged with SiREN resulting in potential recall bias. However, this longer-term follow-up enabled the identification of outcomes that would not have been distinguishable immediately after engagement had occurred.

## Conclusion

This study used causal loop diagrams to provide new insight into how a partnership-based project contributed to building research and evaluation capacity. Findings suggest a complex interplay of contextual and process factors promoted engagement with SiREN, which resulted in research, evaluation, and evidence-informed decision-making capacity improvements within the system. The use of causal loop diagrams highlighted key leverage points that may be exploited to facilitate improvement and evaluation. The focus on contextual factors and their relationship to engagement provide valuable guidance for researchers, policymakers or practitioners seeking to develop or evaluate a similar capacity building partnership.

## Data Availability Statement

The raw data supporting the conclusions of this article will be made available by the authors, without undue reservation.

## Ethics Statement

The studies involving human participants were reviewed and approved by Curtin University Human Research Ethics Committee. The patients/participants provided their written informed consent to participate in this study.

## Author Contributions

The study was conceptualized by RT, JH, BM, and RL. RT undertook recruitment, data collection and analysis with input and supervision from RL, JH, and BM. RT drafted and edited the manuscript. RL, JH, BM, and GC provided critical feedback. All authors have approved the final manuscript.

## Funding

This study was undertaken as part of RT doctoral studies. To undertake their doctoral studies RT was supported by an Australian Government Research Training Program Scholarship, a completion scholarship from the Graduate Research School at Curtin University, and a scholarship from SiREN which was supported by the Sexual Health and Blood-Borne Virus Program, Government of Western Australia Department of Health.

## Conflict of Interest

This manuscript presents a study undertaken as part of RT's PhD that examined SiREN. RT has previously been employed by SiREN. RL is the manager of SiREN. GC and JH are on SiREN management team. A scholarship from SiREN was paid to the PhD student (RT). The remaining author declares that the research was conducted in the absence of any commercial or financial relationships that could be construed as a potential conflict of interest.

## Publisher's Note

All claims expressed in this article are solely those of the authors and do not necessarily represent those of their affiliated organizations, or those of the publisher, the editors and the reviewers. Any product that may be evaluated in this article, or claim that may be made by its manufacturer, is not guaranteed or endorsed by the publisher.
